# Real world evidence for glucosamine-chondroitin-calcium supplementation on joint pain

**DOI:** 10.3389/fnut.2026.1888176

**Published:** 2026-09-22

**Authors:** Jingmeng Ju, Zhongxia Li, Jialu Yang, Chaoming Deng, Min Xia

**Affiliations:** 1 Guangdong Provincial Key Laboratory of Food, Nutrition and Health, and Department of Nutrition, School of Public Health, Sun Yat-sen University, Guangzhou, China; 2 BYHEALTH Institute of Nutrition & Health, Guangzhou, China

**Keywords:** calcium, chondroitin sulfate, glucosamine sulfate, joint discomfort, real-world

## Abstract

**Background:**

Joint and bone diseases are major causes of morbidity worldwide. While glucosamine sulfate (GS) and chondroitin sulfate (CS) have demonstrated benefits in severe degenerative joint conditions, real-world evidence on the use patterns and reported outcomes of their combination with calcium (GCC) among individuals with mild-to-moderate joint discomfort remains limited.

**Methods:**

This real-world study enrolled 9,090 adults who used GCC for ≥1 month and completed an online questionnaire; pre- and post-use symptom scores were analyzed among participants reporting joint symptoms or back pain. Linear mixed-effects models adjusted for demographics, lifestyle, comorbidities, medications, and other dietary supplement use.

**Results:**

After GCC use, joint pain, stiffness, swelling, inflexibility and back pain scores were significantly lower than pre-use scores, with mean changes of −1.74 (95% CI: −1.81 to −1.68) for joint pain and −1.37 (95% CI: −1.45 to −1.28) for back pain (all *P* < 0.001), although none of the mean changes reached the pre-specified primary two-point minimal clinically important difference. Notably, intake at or above the recommended dosage was associated with more favorable changes in pain, stiffness, and back pain scores. Longer intake duration, better adherence, and physical activity were also associated with more favorable changes in some symptom scores. Self-reported adverse events were rare (5.61%), predominantly gastrointestinal.

**Conclusion:**

Although the mean changes did not reach the pre-specified primary two-point minimal clinically important difference, GCC use was associated with lower joint discomfort and back pain scores, and self-reported adverse events were rare.

## 1 Introduction

Osteoarthritis (OA), which is a degenerative joint disease that is characterized by the breakdown of cartilage and results in joint pain, stiffness, and compromised mobility, has become a leading cause of disability worldwide in recent decades (1). According to the Global Burden of Disease Study 2021, 595 million people suffered from OA in 2020, accounting for 7.6% of the global population; among these patients, the majority were older adults (2). A sharp increase in OA and its related complications has also been observed in China, where approximately 152.85 million individuals were affected in 2021 (3, 4).

Glucosamine sulfate (GS) is a compound of glucosamine that naturally occurs in the human body, specifically in the fluid around joints (5). GS, which is derived from crustacean chitin, primarily obtained from shrimp and crab shells (6), has been reported to exert a profound effect on alleviating OA through several mechanisms, such as suppressing interleukin-1 and its downstream inflammatory mediators and reducing matrix degradation, all of which are responsible for cartilage breakdown (7, 8). Similarly, chondroitin sulfate (CS), which is a key component of the extracellular matrix in animal cartilage, has anti-inflammatory effects and promotes chondroprotection by regulating cartilage metabolism and inflammatory responses in the osteoarticular system (9, 10). Consistent with observations in animal models, clinical trials involving patients with OA demonstrated that, compared with placebo, GS significantly slowed joint space narrowing and alleviated OA-related symptoms (11), whereas CS substantially reduced cartilage volume loss and bone marrow lesions (12). Moreover, compared with placebo, a notable reduction in joint space narrowing has also been reported in patients with OA who took a combination of GS and CS (13). Given the potential benefits for alleviating the symptoms associated with OA, GS and CS have gained high popularity in the public, but ongoing controversy and mixed clinical evidence about their efficacy remain. A recent meta-analysis demonstrated an inconsistent conclusion about the effectiveness of glucosamine, possibly due to the fact that most trials were conducted in patients with OA who presented with moderate to severe joint pain and typically included a relatively short duration (typically less than 6 months) (14–18). Moreover, population-based data suggest that glucosamine use is not confined to individuals who report arthritis (19). To date, however, evidence regarding longer-duration use, user-reported symptoms, and self-reported adverse events among consumers without OA remains limited. In particular, the real-world use patterns and reported outcomes of GS- and CS-containing supplements among individuals with mild or non-specific musculoskeletal symptoms, as well as among those using these products for preventive purposes, remain insufficiently characterized.

In contrast to randomized controlled trials (RCTs), which are designed to establish causal efficacy under tightly controlled conditions, real-world evidence (RWE) studies aim to evaluate how interventions perform in routine practice among broader, more heterogeneous populations (20). RWE reflects actual adherence patterns, concomitant treatments, and patient behaviors that are often excluded from traditional trials, thus offering insights into the effectiveness and generalizability of interventions in everyday use. Accordingly, real-world studies complement RCTs by translating trial-based findings into practical, population-level contexts rather than replicating controlled comparisons. In this context, our study was designed as a real-world evaluation of a widely used dietary supplement containing glucosamine, chondroitin sulfate, and calcium (GCC). Rather than focusing on causal inference against a placebo control, we sought to characterize patterns of GCC use and user-reported symptom changes under real-life conditions, thereby generating real-world evidence that complements findings from controlled clinical trials and provides insights into everyday consumer use. The distinctive contribution of this study lies in its combined assessment of dosage, intake frequency, duration, adherence, retrospectively reported joint and back symptom changes, individual improvement based on a pre-specified minimal clinically important difference, and self-reported adverse events within a large sample of GCC users. To our knowledge, this is the first large-scale real-world study to examine these features together among GCC users.

## 2 Materials and methods

### 2.1 Study design and participants

This retrospective cohort study was conducted across China from May 2022 to March 2024. Adults who had taken the supplement Jianliduo^®^, which consists of GS, CS, and calcium (GCC), for at least 1 month were voluntarily recruited through online posters and flyers and invited to complete an online survey. The supplements were produced by BYHEALTH Co., Ltd (Guangzhou, China). Individuals with mental illness that rendered them unable to understand the nature, scope, and possible consequences of the study were excluded. The study was approved by the Ethics Committee of the School of Public Health, Sun Yat-sen University (2021-064), and it was conducted in accordance with the principles of the Declaration of Helsinki. Written informed consent was obtained from all the participants.

A total of 10,220 individuals responded to our survey, and 1,130 individuals were excluded due to the following reasons: (1) missing information about gender (*n* = 880); (2) missing information about age or reporting an unreasonable age (*n* = 88); (3) missing information about the duration of GCC supplement use (*n* = 51); and (4) extreme outliers of body mass index (BMI, *n* = 111, Supplementary Figure 1). Specifically, BMI was calculated based on self-reported height and body weight, and a reported BMI value greater than 65 kg/m^2^ or less than 16 kg/m^2^, height above 210 cm or below 120 cm, and weight above 180 kg or below 35 kg were considered implausible, and thus, these subjects were excluded from further analysis (21, 22). Among the remaining 9,090 participants, 6,224 reported using the GCC supplement for joint- or bone-related purposes, while 2,866 reported taking it without current joint or back symptoms and described their use as preventive. The preventive-use group was not included in the symptom analyses. Ultimately, 3,896 participants were included in the joint symptom analysis and 2,388 in the back pain analysis.

### 2.2 Assessment of self-reported joint discomfort and back pain

Both pre- and post-use ratings of joint discomfort and back pain were collected in the same online questionnaire using investigator-developed 0–10 rating items, for which formal reliability and validity had not been established. The pre-use ratings were based on participants’ retrospective recall. The symptom domains were selected with reference to established tools, including the Western Ontario and McMaster Universities Osteoarthritis Index (WOMAC) and the Knee injury and Osteoarthritis Outcome Score (KOOS). Both the Chinese versions of WOMAC and KOOS have been culturally adapted and psychometrically validated in mainland Chinese populations, demonstrating good internal consistency and test-retest reliability (23, 24). The questionnaire focused on four key dimensions of joint health, including pain, stiffness, swelling, and inflexibility. A self-reported visual analog scale (VAS) ranging from 0 to 10 was used to evaluate the severity of each symptom. For joint pain, stiffness, and swelling, 0 indicated no symptoms, and 10 indicated extremely severe symptoms, whereas for inflexibility, 0 indicated ideal flexibility, and 10 indicated extreme inflexibility. In addition to joint-related symptoms, back pain, typical manifestations of osteoporosis, was also assessed using similar VAS scales, with 0 indicating no pain at all and 10 indicating severe pain. Lower post-use than recalled pre-use scores indicated lower reported symptom severity. The complete set of questionnaire items is shown in Supplementary Table 1.

### 2.3 Assessment of the intake of the GCC supplement

The frequency, duration, and dosage of GCC supplement use were collected by an online questionnaire. The participants were asked to report the frequency with which they took the GCC supplement, with options ranging from daily to less than once a week. Considering the recommended dosage of the GCC supplement, subjects were classified into two groups according to their daily intake dosage: the 1–2 tablet group (half of the recommended dose) and the 3–4 tablet group (recommended dose, corresponding to 535.5–714 mg/day of GS, 306–408 mg/day of CS and 275–367 mg/day of calcium). Due to the limited sample size, subjects with a daily intake of more than four tablets were combined and included in the 3–4 tablets or higher group (recommended or higher dose).

### 2.4 Assessment of other variables

Demographic characteristics, clinical history, medication use, and lifestyle factors, including alcohol consumption, smoking status, self-reported dietary supplements, and physical activity, were collected via questionnaires. Current smoking habits were defined as the use of tobacco products at the time of the survey (25). Alcohol consumption was defined as having consumed alcoholic beverages at least once a week over the past year at the time of the survey (26). Physical activity was assessed based on questions about the frequency of physical activity lasting 30 min or more during leisure time. Given the overall low activity level in the study population, participants were categorized as physically active if they engaged in physical activity at least once a week. Regular dairy consumption was defined as consuming dairy products at least three times per week, regardless of the type. The use of dietary supplements other than the GCC supplement that are beneficial for joint health or osteoporosis was also recorded. Joint health supplements were defined as products containing glucosamine, chondroitin sulfate, curcumin, hyaluronic acid, or collagen, whereas osteoporosis supplements were defined as those containing calcium, vitamin D, or vitamin K. Moreover, medical history and medication use were collected. Participants were asked whether they had previously received a physician diagnosis of osteoarthritis, rheumatoid arthritis, gouty arthritis, or osteoporosis. These diagnoses were self-reported. Medication history primarily covered treatments for osteoporosis, joint-related disorders (including osteoarthritis, psoriatic arthritis, rheumatoid arthritis, and gouty arthritis), or other chronic non-communicable diseases.

### 2.5 Definition and evaluation of symptom relief

To evaluate the clinical magnitude of changes in VAS scores, both anchor-based and distribution-based approaches were considered when selecting the minimal clinically important difference (MCID). For the anchor-based method, two studies in knee osteoarthritis reported MCID thresholds of approximately 9 and 19.9 mm on a 0–100 mm VAS (27, 28) for joint pain, corresponding to roughly 1–2 points on a 0–10 scale. For the distribution-based approach, we applied 0.5× the standard deviation (SD) of the retrospectively reported pre-use symptom scores, following the widely used “half-standard deviation” rule for identifying clinically meaningful change (29). The resulting estimates ranged from 1.19 to 1.49 points across symptoms, which fell within the same range as the anchor-based thresholds (Supplementary Table 2). Because established MCID values are unavailable for the investigator-developed non-pain symptoms items (swelling, stiffness, flexibility, and back pain), the distribution-based estimates were adopted as empirical guidance for selecting a common threshold across outcomes. Given the integer nature of our 0–10 scale, we pre-specified a two-point change as the primary MCID threshold. For consistency, improvement was defined as a reduction of ≥2 points, and a one-point change was additionally examined only as a sensitivity threshold and was not used for the primary clinical interpretation. Moreover, for a more comprehensive evaluation of joint health, we introduced a composite metric, namely, overall joint symptom. For this composite outcome, an individual was considered to have achieved improvement if at least one of the four joint-related symptom scores decreased by ≥2 points.

### 2.6 Study endpoints and analytic hierarchy

The primary endpoint of this study was the change in joint pain score, defined as the difference between the post-use score and the retrospectively recalled pre-use score. Secondary endpoints included the changes in joint stiffness, swelling, inflexibility, and back pain, as well as differences in symptom-score changes according to dose and duration of use. In addition, exploratory analyses were performed to assess whether the reported symptom changes differed across subgroups defined by adherence level, gender, age, physical activity, and the number of joints affected.

### 2.7 Assessment of self-reported adverse events

Adverse events (AEs) were collected through retrospective questionnaires. As dietary supplements currently lack a dedicated pharmacovigilance system and the real-world design precluded precise capture of AE onset times, exposure-adjusted incidence rates could not be calculated in the strict sense. Instead, time-standardized AE proportions were computed, defined as the number of participants reporting ≥ 1 AE divided by the total person-months of GCC use. For participants reporting multiple AEs, the total number of events was also standardized by person-months to yield event rates per person-month. These measures approximate exposure-adjusted reporting rates under real-world conditions. In addition, causality between GCC use and reported AEs was classified according to the World Health Organization-Uppsala Monitoring Centre (WHO-UMC) causality categories.

### 2.8 Statistical analysis

The basic characteristics of the study participants were summarized according to gender as the mean ± SD or median (interquartile range) for continuous variables and *n* (%) for categorical variables. Independent Student’s *t*-test was used to compare normally distributed variables, the Mann-Whitney U test was used to compare variables with highly skewed distributions, and the χ2 test was used to compare categorical variables. For the analysis of symptom relief rates, proportions were reported along with their corresponding 95% confidence intervals (CIs), which were calculated using the Wilson score method.

In the main analysis, linear mixed-effects models were used to compare pre-use and post-use joint discomfort and back pain scores among GCC users, with a within-subject comparison (pre- and post-use). A random intercept at the individual level was included to account for the correlation between repeated measures within participants and to allow each participant to have an individual-level symptom level. The models were fitted using the lme4 package in R software (version 1.1.35.5). Potential confounders were identified based on directed acyclic graphs (Supplementary Figure 2) illustrating the presumed relationships among the study variables, and all selected confounders relevant to the observed within-user symptom-score differences were fully adjusted for in the models. These included time (pre- and post-use), demographic characteristics (age, gender, BMI and educational attainment), medical history of arthritic conditions or osteoporosis, GCC supplement dosage, duration, and frequency, intake of other joint or bone health supplements, lifestyle factors (current smoking, regular dairy intake, and physical activity), and medication use (antihypertensive, antidiabetic, and antihyperlipidemic drugs). Several subgroup analyses, stratified by intake dosage, duration, adherence, gender, age, number of affected joints, and regular physical activity, were conducted. In these stratified analyses, differences in joint discomfort and back pain score changes across groups were assessed using linear mixed-effects models. The fixed effects in the model included group, time (pre- and post-use), group-by-time interaction, and covariates, including age, gender, BMI, educational attainment, self-reported arthritic conditions/osteoporosis, daily GCC supplement dosage, intake duration, intake frequency, use of other joint/bone-health supplements, current smoking habits, regular dairy intake, physical activity, the use of antihypertensive, antidiabetic, and antihyperlipidemic medications, as well as the corresponding pre-use symptom score of each participant. Pairwise comparisons were conducted using linear contrasts to estimate between-group differences in reported symptom-score changes.

To ensure the robustness of our findings, several sensitivity analyses were conducted. The propensity score (PS) (30) was calculated using multivariable logistic regression to account for potential confounding by measured covariates across subgroups. Three PS methods were used to improve the balance of measured covariates between comparison groups: (1) nearest neighbor matching at a 1:1 ratio with a caliper of 0.1 to identify comparison pairs across subgroups; (2) inverse probability of treatment weighting (IPTW) using stabilized weights to estimate the average treatment effect (ATE), with extreme weights trimmed at the 1st and 99th percentiles; and (3) overlap weighting (OW) (31), which assigned greater weight to participants with greater covariate overlap between the comparison groups. For each subgroup analysis, a separate PS model tailored to the specific comparison was constructed. The covariate balance between subgroups was evaluated using standardized mean differences (SMDs), accounting for the weights. A standardized difference of less than 0.1 was considered acceptable, indicating adequate covariate balance after applying PS. When performing subgroup analyses stratified by a given variable (such as intake duration or dosage), the corresponding variable was excluded from the list of covariates adjusted to avoid overadjustment. Nearest neighbor matching was performed using the MatchIt package (v4.5.5) in R, whereas IPTW and OW were performed using the WeightIt package (v1.3.0). More details about the PS models are provided in the Supplementary material.

All the data analyses were performed using R version 4.4.1 (The R Foundation for Statistical Computing, Vienna, Austria), and a two-sided *P* < 0.05 was considered to indicate a statistically significant difference.

## 3 Results

### 3.1 Characteristics of the study participants

Among the 9,090 participants who were included in this study, the average age of the study participants was 48.53 years, and men were significantly older than women. Self-reported physician-diagnosed gouty arthritis was more prevalent in men (2.43%) than in women (0.88%, *P* < 0.001), whereas osteoporosis was more common in women (19.55%) than in men (17.73%, *P* = 0.045). There were no significant gender differences in the prevalence of osteoarthritis, rheumatoid arthritis, or other comorbidities. Men were more likely to use medications but also had more unhealthy lifestyle behaviors. The use of joint health and osteoporosis supplements was significantly greater among women than among men (*P* < 0.001 for both). Moreover, in addition to the GCC supplement, more than half of the participants took other osteoporosis-beneficial supplements (Table 1). Among participants with joint symptoms, 24.5% reported physician-diagnosed osteoarthritis, 1.9% rheumatoid arthritis, and 1.8% gouty arthritis, whereas 73.5% reported none of these diagnoses. Among those with back pain, 34.5% reported physician-diagnosed osteoporosis (Supplementary Table 3).

**TABLE 1:** Characteristics of the study participants.

Characteristics	Total (*n* = 9,090)	Women (*n* = 6,240)	Men (*n* = 2,850)	*P*-value
Demographics				
Age, y	48.53 ± 13.18	47.91 ± 13.00	49.90 ± 13.47	<0.001
BMI, kg/m^2^	24.43 ± 5.45	24.25 ± 5.66	24.82 ± 4.95	<0.001
Educational attainment, *n* (%)				
Primary or below	1,120 (15.4)	760 (15.4)	360 (15.4)	0.577
Middle or high school	3,840 (52.7)	2,585 (52.3)	1,255 (53.5)	–
College or above	2,322 (31.9)	1,593 (32.3)	729 (31.1)	–
Comorbidities				
Osteoarthritis, *n* (%)	1,312 (14.68)	884 (14.38)	428 (15.32)	0.260
Rheumatoid arthritis, *n* (%)	111 (1.24)	85 (1.38)	26 (0.93)	0.091
Gouty arthritis, *n* (%)	122 (1.36)	54 (0.88)	68 (2.43)	<0.001
Osteoporosis, *n* (%)	1,703 (18.98)	1,205 (19.55)	498 (17.73)	0.045
Medical history				
Joint surgery, n (%)	256 (2.85)	176 (2.85)	80 (2.85)	0.990
Anti-inflammatory and analgesic drugs, *n* (%)	1,299 (14.48)	861 (13.97)	438 (15.60)	0.046
Medications for relieving joint swelling, *n* (%)	299 (3.33)	216 (3.50)	83 (2.96)	0.201
Anti-osteoporosis drugs, *n* (%)	466 (5.19)	298 (4.84)	168 (5.98)	0.026
Estrogen therapy, *n* (%)	39 (0.43)	29 (0.47)	10 (0.36)	0.555
Antihypertensive drugs, *n* (%)	881 (9.82)	521 (8.45)	360 (12.82)	<0.001
Antidiabetic drugs, *n* (%)	338 (3.77)	207 (3.36)	131 (4.67)	0.003
Antihyperlipidemic drugs, *n* (%)	365 (4.07)	214 (3.47)	151 (5.38)	<0.001
Lifestyles				
Drinking, *n* (%)	637 (8.75)	98 (1.99)	539 (22.99)	<0.001
Current smoking, *n* (%)	1,408 (19.33)	201 (4.07)	1,207 (51.45)	<0.001
Regular dairy intake, *n* (%)	2,450 (33.68)	1,887 (38.22)	563 (24.09)	<0.001
Physically active, *n* (%)	3,428 (47.19)	2,322 (47.13)	1,106 (47.31)	0.907
Other joint health supplements, *n* (%)	1,714 (19.09)	1,336 (21.66)	378 (13.45)	<0.001
Other osteoporosis health supplements, *n* (%)	4,902 (54.59)	3,549 (57.53)	1,353 (48.15)	<0.001

Data were expressed as mean ± SD, or *n* (%). *P*-values were determined by Student’s *t*-test for continuous variables and the chi-square test for categorical variables. BMI, body mass index.

Only 36.90% of the study participants used the GCC supplement primarily for joint discomfort relief; 17.09% of them took the GCC supplement specifically for back pain; 14.48% of them used the GCC supplement for both joint and bone discomfort; and 31.53% of the participants reported no current joint or back symptoms and described their use as preventive (Supplementary Figure 3A). In terms of dosage, more than 60% of the participants followed the recommended intake of 3–4 tablets daily, whereas 37.43% of them took a lower dose of 1–2 tablets per day (Supplementary Figure 3B). In terms of frequency, more than 90% of the participants took the GCC supplement at least three times per week (Supplementary Figure 3C). The median duration of GCC supplement intake was 3 months. At the time of the survey, 21.50% of the participants had been taking the GCC supplement for at least 6 months (Supplementary Figure 3D).

### 3.2 Changes in joint discomfort and back pain scores

Among the 3,896 participants who reported taking the GCC supplement for joint discomfort relief, after adjusting for time (pre- and post-use), demographic characteristics, medical history, dosage, duration, lifestyle factors, dietary supplement use, and medication history, all the symptoms of joint discomfort, including pain, stiffness, and swelling, showed significantly lower post-use scores than pre-use scores (−1.74, 95% CI: −1.81 to −1.68 for pain; −1.42, 95% CI: −1.49 to −1.36 for stiffness; and −1.04, 95% CI: −1.11 to −0.98 for swelling, respectively; Figure 1). Although these mean changes exceeded the one-point sensitivity threshold, they did not reach the predefined primary two-point MCID, suggesting that the average magnitude of the reported changes was modest. Of note, the standardized response means (SRMs) for the three symptoms (pain, stiffness and swelling) ranged from −0.80 to −0.51 (Supplementary Table 4), reflecting moderate-to-large responsiveness of the symptom scores. For joint inflexibility, a smaller yet significant change was also observed (−0.67, 95% CI: −0.77 to −0.57), which did not reach either the one-point sensitivity threshold or the primary two-point MCID. Among the 2,388 participants who reported taking the GCC supplement primarily to relieve back pain, the mean change (−1.37, 95% CI: −1.45 to −1.28; Figure 1) similarly exceeded the one-point sensitivity threshold but not the primary two-point threshold, with an SRM of −0.66, indicating moderate responsiveness (Supplementary Table 4). Notably, the changes in joint discomfort and back pain scores remained materially unchanged among participants experiencing both conditions (Supplementary Figure 4).

**FIGURE 1:**
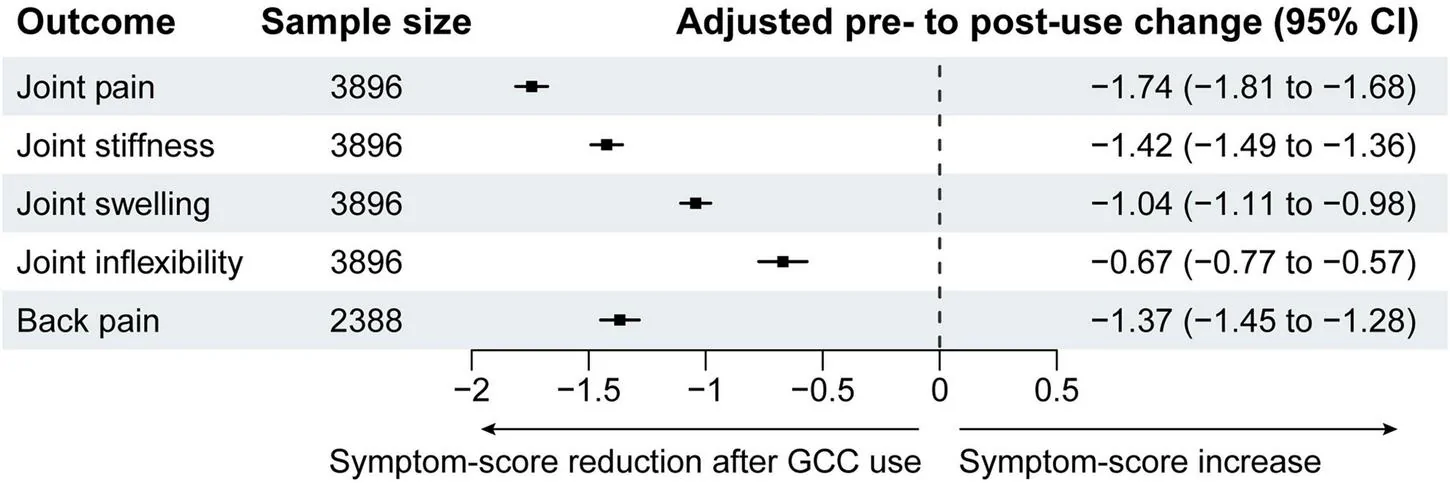
Changes in joint discomfort and back pain scores before and after GCC use. The estimates were obtained from linear mixed-effects models, adjusting for time (pre- and post-use), age, gender, BMI, educational attainment, self-reported arthritic conditions or osteoporosis, daily GCC supplement dosage, intake duration, intake frequency, use of other joint- or bone-benefiting supplements, smoking status, regular dairy intake, physical activity, and the use of antihypertensive, antidiabetic, and antihyperlipidemic medications.

### 3.3 Changes in symptom scores across dosage and duration groups

To further compare changes in symptom scores across different dosages and duration groups, group and group-by-time interaction terms were further included in the linear mixed-effects models. After full adjustment, taking three or more tablets per day, for which subjects were included in the recommended or higher dosage group, was associated with greater reductions in joint pain (−0.19, 95% CI: −0.27 to −0.10), stiffness (−0.12, 95% CI: −0.21 to −0.03), and back pain (−0.39, 95% CI: −0.49 to −0.28) than taking a half dose of 1–2 tablets per day (Figure 2). This observation remained largely the same in several sensitivity analyses conducted in well-balanced subgroups (Supplementary Figures 5, 6 and Supplementary Table 5), suggesting that intake at or above the recommended dosage may be associated with more favorable changes in joint discomfort and back pain than intake below the recommended dosage. In contrast, compared with a short duration of 1–3 months, longer durations of GCC supplement intake were associated with greater reductions in joint symptoms, including pain, stiffness, swelling, and inflexibility (Figure 2). Similarly, duration-related patterns in joint symptom-score changes were observed in several sensitivity analyses (Supplementary Figures 5, 6 and Supplementary Table 5), further suggesting that longer-duration GCC use may be associated with more favorable changes in joint symptoms. For back pain, no obvious association between intake duration and score changes was observed, possibly due to the limited number of participants who took the GCC supplement for this purpose (Figure 2 and Supplementary Figures 5, 6).

**FIGURE 2:**
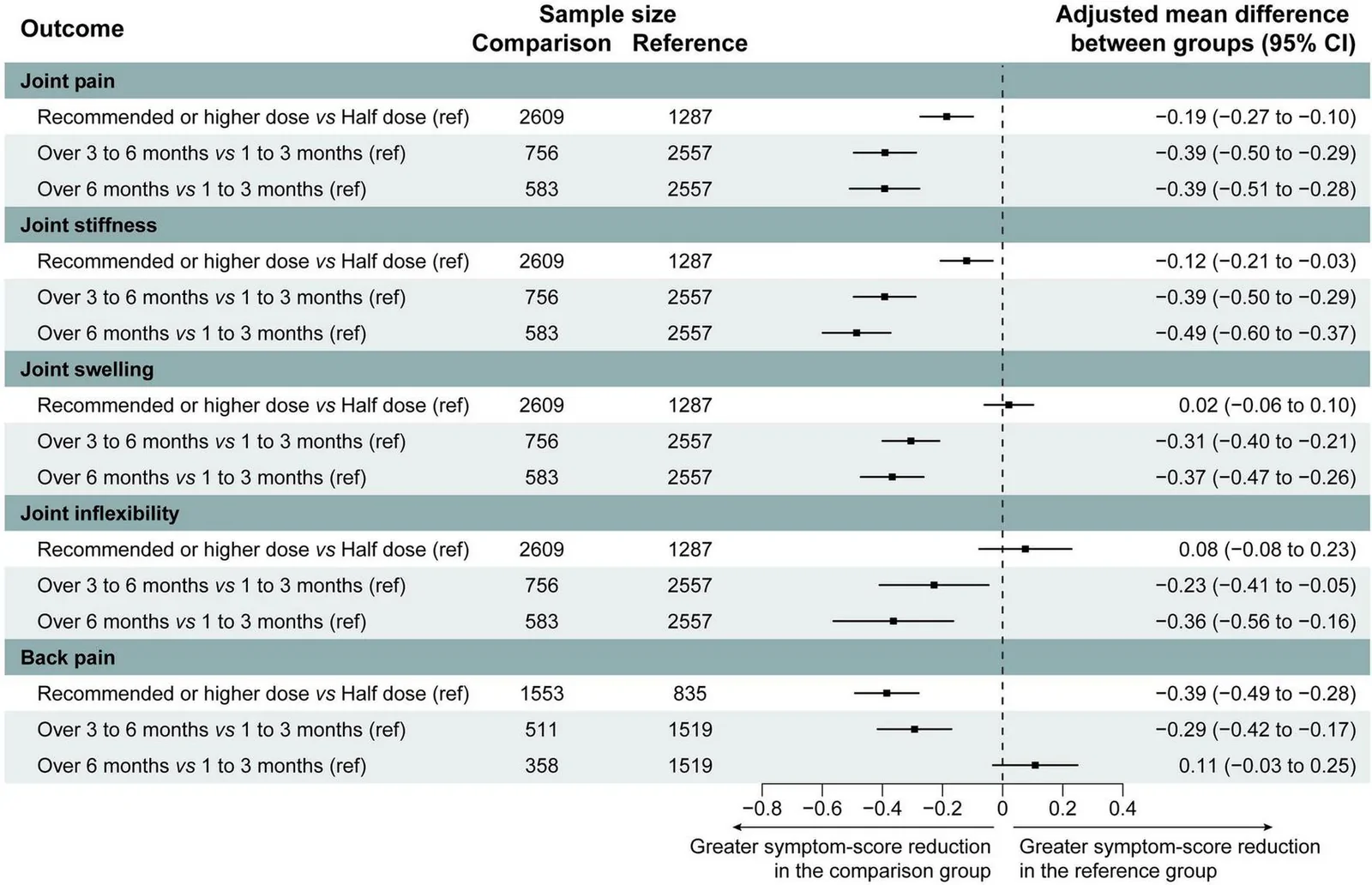
Changes in joint discomfort and back pain scores by glucosamine, chondroitin sulfate, and calcium (GCC) dosage and duration of use. Differences in joint discomfort and back pain score changes were analyzed across subgroups stratified by intake dosage and duration. The estimates were derived from linear mixed-effects models, adjusting for group, time (pre- and post-use), group-by-time interaction, age, gender, BMI, educational attainment, self-reported arthritic conditions or osteoporosis, daily GCC supplement dosage, intake duration, intake frequency, use of other joint or bone health supplements, current smoking habits, regular dairy intake, physical activity, use of antihypertensive, antidiabetic, and antihyperlipidemic medications, as well as the corresponding pre-use symptom score for each participant. To avoid overadjustment, the variable used for each subgroup stratification, such as dosage or intake duration, was not included as a covariate in the corresponding model.

To further explore whether the observed score changes reached the pre-specified threshold for clinically meaningful improvement, the MCID for each symptom was used (primary threshold: a reduction of ≥2 points), and the proportion of participants meeting this threshold was assessed. Among participants who took a half-dose of 1–2 tablets per day, the proportions meeting the primary threshold for overall joint symptoms and back pain were 58.74% (95% CI: 55.99% to 61.44%) and 27.54% (95% CI: 24.57% to 30.73%), respectively (Table 2). In contrast, among those taking at least three tablets per day, the corresponding proportions were 70.83% (95% CI: 69.04% to 72.56%) and 44.75% (95% CI: 42.26% to 47.27%), respectively. Notably, proportions meeting the primary threshold tended to increase with increasing intake duration, regardless of the dosage taken per day (Table 2). Conversely, across all the intake durations, participants who took at least three tablets per day consistently demonstrated higher proportions meeting the primary threshold for overall joint symptoms and back pain than those taking a half-dose (Table 2), suggesting that intake at or above the recommended dosage and longer-duration use may be associated with more favorable individual symptom-score changes. The results remained consistent when the one-point sensitivity threshold was used, although this threshold was not used for the primary clinical interpretation (Supplementary Table 6).

**TABLE 2:** Proportions with clinically meaningful symptom-score reduction (≥2-point MCID threshold) by dose and duration.

Outcome	Relief rates based on MCID ≥ 2 (95% CI, %)				
	All	1 month	>1 to ≤3 months	>3 to ≤6 months	>6 months
Half dosage					
Joint pain	39.16 (36.49 to 41.90)	28.77 (23.66 to 34.46)	36.02 (32.06 to 40.18)	48.67 (42.02 to 55.37)	50.92 (44.10 to 57.70)
Joint stiffness	31.62 (29.10 to 34.26)	22.11 (17.52 to 27.46)	27.42 (23.8 to 31.36)	38.50 (32.18 to 45.21)	47.71 (40.95 to 54.55)
Joint swelling	25.10 (22.77 to 27.58)	15.79 (11.86 to 20.67)	21.15 (17.88 to 24.82)	33.19 (27.17 to 39.79)	38.99 (32.54 to 45.83)
Joint inflexibility	27.51 (25.10 to 30.05)	19.3 (14.98 to 24.46)	27.24 (23.63 to 31.17)	33.19 (27.17 to 39.79)	33.03 (26.91 to 39.75)
Overall joint symptom	58.74 (55.99 to 61.44)	47.37 (41.47 to 53.34)	55.38 (51.14 to 59.54)	66.81 (60.21 to 72.83)	73.85 (67.40 to 79.45)
Back pain	27.54 (24.57 to 30.73)	19.77 (14.33 to 26.56)	26.37 (22.09 to 31.14)	41.33 (33.45 to 49.67)	25.60 (18.41 to 34.32)
Recommended or higher dosage					
Joint pain	54.47 (52.53 to 56.39)	45.53 (40.46 to 50.68)	54.5 (51.78 to 57.19)	58.49 (54.15 to 62.70)	57.81 (52.55 to 62.90)
Joint stiffness	43.96 (42.05 to 45.89)	34.74 (30.00 to 39.79)	42.8 (40.14 to 45.51)	51.32 (46.98 to 55.64)	47.12 (41.92 to 52.38)
Joint swelling	30.66 (28.90 to 32.48)	20.53 (16.65 to 25.01)	29.16 (26.75 to 31.69)	38.68 (34.54 to 42.99)	35.07 (30.22 to 40.24)
Joint inflexibility	31.62 (29.85 to 33.45)	23.68 (19.56 to 28.35)	31.26 (28.79 to 33.84)	35.47 (31.43 to 39.73)	35.62 (30.75 to 40.80)
Overall joint symptom	70.83 (69.04 to 72.56)	58.95 (53.8 to 63.91)	70.09 (67.54 to 72.52)	77.36 (73.51 to 80.81)	76.44 (71.68 to 80.63)
Back pain	44.75 (42.26 to 47.27)	33.52 (26.7 to 41.08)	44.7 (41.19 to 48.26)	50.42 (45.14 to 55.68)	44.64 (38.18 to 51.27)

MCID, minimal clinically important difference.

### 3.4 Changes in symptom scores across different subgroups

Moreover, a series of subgroup analyses were performed to compare changes in symptom scores across different subgroups. As expected, after adjusting for potential confounders, greater adherence based on the categorical additive score was associated with greater reductions in the scores for all types of joint- and bone-related discomfort, including joint pain (−0.48, 95% CI: −0.57 to −0.40), stiffness (−0.43, 95% CI: −0.51 to −0.34), swelling (−0.25, 95% CI: −0.33 to −0.17), joint inflexibility (−0.35, 95% CI: −0.49 to −0.20), and back pain (−0.44, 95% CI: −0.55 to −0.34; Figure 3). Similar associations were observed when adherence was evaluated using the continuous standardized additive (z-add) measure (Figure 3), supporting the robustness of the findings. Compared with participants aged 50 years or younger, those older than 50 years showed greater reductions in the scores for joint pain (−0.20, 95% CI: −0.29 to −0.11), stiffness (−0.18, 95% CI: −0.27 to −0.09), swelling (−0.15, 95% CI: −0.23 to −0.07), and back pain (−0.14, 95% CI: −0.25 to −0.04) but a smaller reduction in joint inflexibility (0.29, 95% CI: 0.14 to 0.45; Figure 3). Additionally, men exhibited greater reductions in the scores for joint stiffness (−0.11, 95% CI: −0.21 to −0.02) and back pain (−0.14, 95% CI: −0.26 to −0.01) but a smaller reduction in joint inflexibility (0.30, 95% CI: 0.13 to 0.47) than women (Figure 3).

**FIGURE 3:**
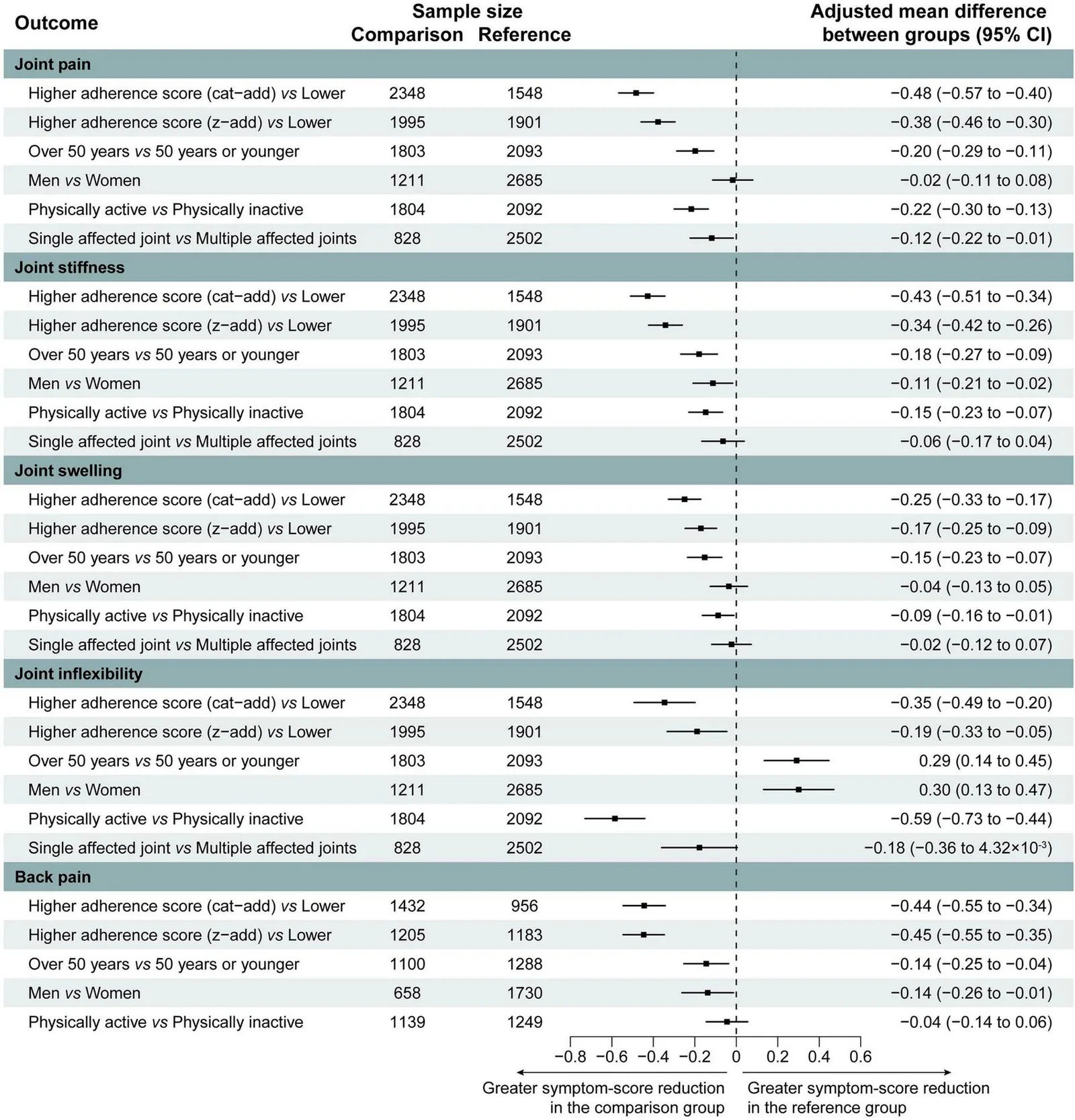
Subgroup differences in joint discomfort and back pain score changes. Differences in joint discomfort and back pain score changes were analyzed across subgroups stratified by adherence score, age, gender, physical activity, and number of affected joints. The estimates were derived from linear mixed-effects models, adjusting for group, time (pre- and post-use), group-by-time interaction, age, gender, BMI, educational attainment, self-reported arthritic conditions or osteoporosis, daily GCC supplement dosage, intake duration, intake frequency, use of other joint or bone health supplements, current smoking habits, regular dairy intake, physical activity, use of antihypertensive, antidiabetic, and antihyperlipidemic medications, as well as the corresponding pre-use symptom score for each participant. To avoid overadjustment, the corresponding stratification variable was not included as a covariate in the model.

Furthermore, given the well-established benefits of physical activity for musculoskeletal health (32, 33), we then compared joint symptom-score changes between physically active and inactive participants. As expected, greater reductions in the scores for joint pain (−0.22, 95% CI: −0.30 to −0.13), stiffness (−0.15, 95% CI: −0.23 to −0.07) and swelling (−0.09, 95% CI: −0.16 to −0.01) and greater improvements in joint inflexibility (−0.59, 95% CI: −0.73 to −0.44) were observed in physically active individuals than in inactive individuals (Figure 3). In contrast, except for joint pain, no significant differences in other joint discomfort score changes were observed between participants with multiple affected joints and those with a single affected joint (Figure 3).

### 3.5 Self-reported AEs during GCC use

Adverse events were relatively rare, and they were reported by 5.61% of the study participants. Constipation was the most commonly reported event (3.31%, 7.74 events per 1,000 person-months), followed by diarrhea (0.92%, 2.23 events per 1,000 person-months), nausea (0.46%) and allergic reactions (0.31%). Insomnia (0.36%) and other or unspecified events (1.18%) were less common (Figure 4 and Supplementary Table 7). According to the WHO-UMC causality assessment, most adverse events were categorized as possible, whereas insomnia and other or unspecified symptoms were generally considered unlikely or unclassified.

**FIGURE 4:**
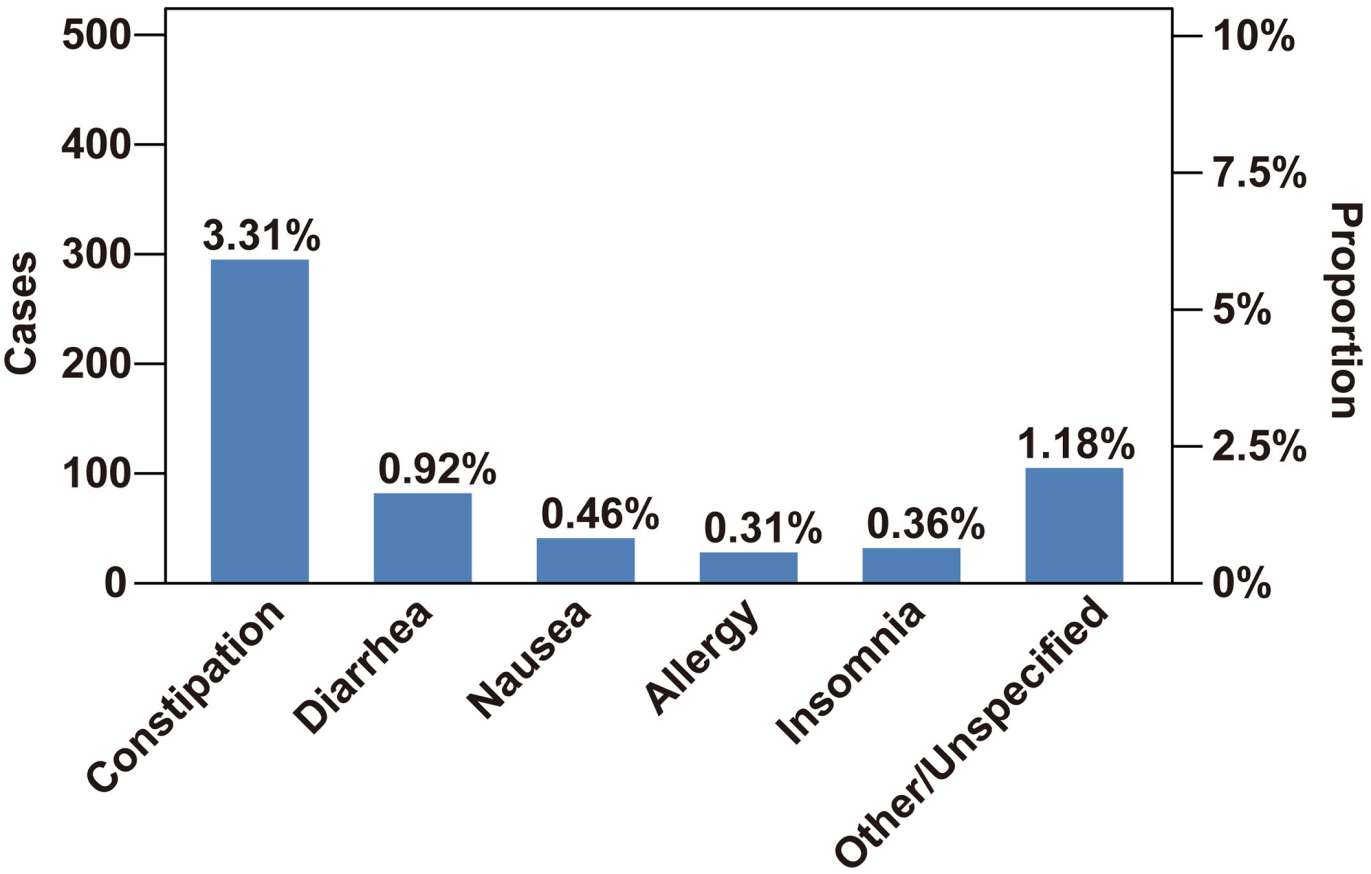
Self-reported adverse events during glucosamine, chondroitin sulfate, and calcium (GCC) use. The number and proportion of self-reported adverse events and their proportions among all participants. The Y-axis is scaled to a maximum of 10%.

## 4 Discussion

This large cohort study in a real-world setting showed that GCC users reported lower post-use joint and back symptom scores than pre-use scores. However, none of the mean symptom-score changes reached the pre-specified primary two-point MCID, indicating that the average magnitude of the reported changes was modest, although some participants achieved reductions of at least two points. The recommended intake of 3–4 GCC tablets per day provided 535.5–714 mg/day of GS, 306–408 mg/day of CS, and 275–367 mg/day of calcium. Intake at or above this level was associated with more favorable changes in joint discomfort and back pain scores than intake below the recommended dosage. Among participants taking at least three tablets per day, the proportions meeting the primary threshold for overall joint symptoms and back pain reached 70.83% and 44.75%, respectively. Longer-duration use of the GCC supplement was associated with more favorable changes in joint symptom scores, and self-reported adverse events were relatively uncommon.

In contrast to well-controlled clinical trials involving patients with severe degenerative diseases, such as patients with Kellgren and Lawrence grade 2–3 OA, which is characterized by moderate-to-severe pain (15, 16), our real-world study did not limit participants to those diagnosed with OA but included all individuals who took the GCC supplement. Notably, only 14.68% and 18.98% of the participants in our study reported osteoarthritis and osteoporosis, respectively, better reflecting the characteristics of the consumers of GS and CS in the real world and providing an opportunity to characterize reported symptom changes among individuals who were not restricted to a specific clinical diagnosis. Previous RCTs investigating GS and CS have yielded inconsistent findings, and the evidence remains controversial (14–16). Differences in study populations and designs may partly contribute to differences between previous RCT findings and our real-world observations. Participants in the RCTs had established structural joint damage, where short-term supplementation is unlikely to reverse radiographic changes or relieve chronic pain. In contrast, the individuals in our real-world study mainly had mild joint discomfort, representing an earlier, potentially reversible stage of joint degeneration. The observed symptom-score changes therefore provide real-world evidence on reported outcomes among GCC users in preventive or early-intervention contexts. To the best of our knowledge, our study is the first large-scale real-world study to jointly characterize GCC use patterns, retrospectively reported symptom changes, and self-reported adverse events among consumers in China. Other previous RCTs typically included only 80∼150 participants in each group, and these participants were allocated to receive GS, CS, or their combination (13, 15). Although the largest RCT to date included 1,583 participants, only 317 individuals were assigned to the group that received a combination of GS and CS (14). Moreover, the evidence base for GS- and CS-containing supplements has been concentrated largely in Europe and North America, whereas direct evidence in Chinese populations remains limited (34). With the rapid rate of population aging, joint symptoms and back pain are becoming prevalent, especially in middle-aged and elderly people. In this context, with a large sample recruited across China, our study provided large-scale real-world evidence on GCC use patterns and reported joint and back symptom changes among Chinese consumers.

GCC is a commercially available combination product with a predetermined formulation, and the exposure assessed in this study was its use under real-world conditions. Glucosamine sulfate and chondroitin sulfate are commonly included in supplements intended for joint health (34), whereas calcium contributes to the maintenance of normal bone health and muscle function (35). Their combination in GCC is consistent with a product intended to address both joint- and bone-health concerns, which may coexist among some older consumers. Although evidence supporting a direct effect of calcium on joint symptoms remains limited, its roles in bone health and normal muscle function may be relevant to back-related outcomes. Because all participants used the combination product, the observed changes in joint- and back-symptom scores cannot be attributed specifically to glucosamine sulfate, chondroitin sulfate, calcium, or any individual component. Future comparative studies may help clarify the contribution of individual components.

Notably, only half of the participants who took the supplement reported joint discomfort, 31.57% reported back pain, and over 30% reported no current joint symptoms or back pain; this distribution reflects a public concern about degenerative joint diseases and a high awareness of prevention. Moreover, in contrast to the strong adherence to prescriptions in clinical trials, approximately 40% of the participants took half of the recommended daily dose, and over 20% of the participants took the GCC supplement fewer than 5 days per week. More importantly, the recommended doses of 535.5–714 mg/day of GS and 306–408 mg/day of CS in the GCC supplement were substantially lower than the standard therapeutic dosages that have been used in RCTs, which were generally 1,500 mg/day for GS and 1,200 mg/day for CS (15). Thus, our findings complemented those of previous RCTs by providing evidence that intake at or above the recommended dosage (GS, 535.5–714 mg/day; CS, 306–408 mg/day) was associated with more favorable changes in joint discomfort and back pain scores among individuals with mild symptoms. Moreover, recommendations regarding GS use remain inconsistent. Some European guidelines or expert statements support the use of GS for selected patients with knee OA, whereas the UK National Institute for Health and Care Excellence does not recommend its use for OA management (36, 37). However, the duration of previous RCTs varied substantially, ranging from 3 to 36 months, complicating the assessment of its long-term safety. In this real-world study we also conducted a comprehensive evaluation of the potential adverse effects during GCC use in the general public. At the recommended dosage, self-reported adverse events were relatively uncommon, with gastrointestinal complaints being the most frequently reported category of events, consistent with previous reports on GS and CS-containing supplements (34). Taken together, these findings suggest that intake of at least three tablets per day may be associated with more favorable reported symptom-score changes than intake below the recommended dosage.

Although statistical significance demonstrated that the observed changes were unlikely to occur due to chance, the MCID was further used to evaluate the clinical significance of these findings (38). Overall, our findings demonstrated that the mean changes in joint pain, stiffness, swelling, inflexibility, and back pain were statistically significant but did not reach the pre-specified primary two-point MCID, indicating that the average clinical magnitude of these changes was modest. Pain, stiffness, swelling, and back pain exceeded the one-point sensitivity threshold, whereas joint inflexibility did not; however, the one-point threshold was not used for the primary clinical interpretation. This discrepancy may be attributed to the fact that functional improvements are often less obvious than pain relief. A study that evaluated the effectiveness of a comprehensive intervention for knee osteoarthritis, which used both the WOMAC and the SF-36, also revealed that pain scores demonstrated the greatest proportional improvement, whereas function-related scores showed relatively smaller changes (39). At the individual level, among participants taking at least three tablets per day, 70.83% achieved a reduction of at least two points in one or more joint symptoms, and 44.75% achieved a reduction of at least two points in back pain. These findings indicate that some participants reported changes meeting the pre-specified primary threshold, despite the modest average changes at the group level. Taken together, these findings suggest statistically significant average symptom-score changes of modest clinical magnitude, with larger changes reported by a subset of participants.

Since OA is a chronic, progressive disease that requires a prolonged period for the evaluation of OA therapy, most RCTs have lasted for no more than 6 months. To examine symptom-score changes associated with different durations of GCC use in individuals with mild joint discomfort, we did not restrict the duration of GCC supplement use in this study. Here, after extensive adjustment, we found that a longer duration of GCC supplement use was associated with greater reductions in the scores for various joint symptoms, including joint pain, stiffness, and swelling. Although we cannot exclude the possibility that individuals with more severe joint discomfort, that is, those who had more room for VAS reduction, were more likely to take the GCC supplement for a longer time, the findings suggest an association between longer-duration GCC use and more favorable changes in joint symptom scores. Moreover, unlike previous analyses that compared dosage and duration separately, an adherence score, which integrates daily dosage, frequency, and duration, was further developed to provide a more comprehensive assessment of participants’ intake behaviors. Notably, a higher adherence score indicated an intake pattern that more closely mirrored those reported in rigorous RCTs. Consistent with better clinical outcomes reported in patients with knee osteoarthritis who had greater adherence to treatment (40), greater reductions in the scores for joint pain, stiffness, swelling, inflexibility, and back pain were also observed in individuals with greater adherence to the recommended use of the GCC supplement than in those with lower adherence. The significant improvement in knee joint function after treatment with a combination of GS and CS in an RCT conducted in Japan (41) was also consistent with our observation of larger reported symptom-score changes among participants with higher adherence scores. Collectively, these findings demonstrated that adherence to the recommended dosage, frequency, and duration of GCC use may be associated with more favorable reported changes in joint and back symptoms.

As a well-established non-pharmacological intervention, exercise has been reported to be effective in improving pain and physical function in individuals with OA (42). However, evidence from human studies on the combined effects of GS/CS supplementation and exercise remains inconsistent (43, 44), which is partially attributed to differences in individual characteristics and severe joint discomfort, which limits exercise in clinical trials. In our study, participants reported relatively mild joint discomfort, and greater reductions in joint pain, stiffness, and inflexibility scores were observed in those who remained physically active, suggesting that regular exercise may complement GCC use and be associated with more favorable symptom-score changes among individuals with mild joint issues. Overall, changes in joint symptom scores were observed across different age and gender groups. However, for specific symptoms, significant differences also existed. For example, individuals older than 50 years showed greater reductions in joint pain, stiffness, and swelling scores, whereas younger individuals showed greater reductions in joint inflexibility scores. Similarly, women showed greater reductions in joint inflexibility scores, whereas men showed greater reductions in joint stiffness scores. This difference might be explained by age- and gender-related differences in joint health and pre-use symptom severity. In contrast, no significant differences in most joint discomfort score changes were observed between individuals with single or multiple affected joints. Overall, symptom-score changes were observed across age, gender, physical activity, and number-of-affected-joints subgroups, supporting the potential relevance of these findings across different groups of GCC users.

In this real-world study, we characterized GCC use and reported symptom-score changes in diverse populations, including both those with and without diagnosed joint conditions. This setting also enabled the consideration of different lifestyle factors and the concurrent use of other medications or dietary supplements. Additionally, we performed comprehensive adjustment for measured confounding factors in the linear mixed-effects model analysis and used three different PS methods to assess the robustness of the observed associations. Compared with previous RCTs on GS- and CS- based supplements, this real-world evidence study offers several complementary insights. First, by including a large real-world sample of GCC users under routine use conditions, our findings support the relevance of the observed associations across users with diverse demographic and health backgrounds. Second, unlike RCTs that typically ensure strict adherence within short intervention windows, our study captured habitual intake behaviors in real-world users, encompassing dosage, frequency, and duration of use, thereby reflecting the natural patterns of supplement consumption in daily life. Third, by covering participants with a wide spectrum of intake durations (≥1 month), our analysis provides novel evidence on longer-duration use patterns and associated symptom-score changes, which have been rarely examined in previous RCTs. Taken together, these features complement existing RCT evidence and provide pragmatic insights into real-world GCC use patterns, adherence, reported symptom changes, and self-reported adverse events.

However, this study also has several limitations. First, the absence of a concurrent control group restricts causal inference. The observed pre- and post-use differences could not be distinguished from natural symptom fluctuations, regression to the mean, concurrent treatments, lifestyle changes, or other unmeasured factors. However, this limitation is common in real-world evaluations, where the objective is to describe outcomes under routine practice rather than establish causal efficacy. RWE thus provides complementary insight into supplement use and reported outcomes among broader, more heterogeneous populations, bridging the gap between clinical trial findings and real-world use. Second, although the present study assessed GCC supplement dosage, frequency, and duration, time-varying confounding cannot be fully ruled out. Future studies adopting longitudinal designs with dynamic collection of both supplement use patterns and time-dependent covariates are warranted to elucidate the relationship between real-world supplement use behaviors and improvement in discomfort symptoms. Third, as a retrospective cohort study, we relied on self-reported questionnaires to assess participants’ joint and back symptoms before and after GCC supplement use, which may introduce recall bias. Participants’ current symptoms and expectations regarding the supplement may have influenced their recalled pre-use and reported post-use scores, potentially exaggerating the observed changes. In addition, the investigator-developed symptom items have not yet undergone formal validation. Future prospective real-world studies incorporating objective measures are warranted to validate and generalize our findings. Fourth, although the selection of covariates for propensity score modeling was generally comprehensive, residual confounding by unmeasured factors such as diet, socioeconomic status cannot be fully excluded. Nevertheless, directed acyclic graphs were used to clarify the potential causal structure and identify plausible confounders, which were subsequently adjusted for in the models. Finally, voluntary online participation may have introduced selection bias, and the absence of information on non-respondents prevented assessment of the response rate and sample representativeness. Therefore, the findings should be generalized cautiously beyond the GCC users who completed this survey.

## 5 Conclusion

In conclusion, GCC use was associated with lower joint discomfort and back pain scores, although the mean changes did not reach the pre-specified primary two-point MCID. Intake at or above the recommended dosage, longer-duration use, and greater adherence may be associated with more favorable symptom-score changes.

## Data Availability

The data that support the findings of this study are available from the corresponding author upon reasonable request.
