# Supplementary material for: Real world evidence for glucosamine-chondroitin-calcium supplementation on joint pain
Source: Front Nutr. 2026 Sep 22;13:1888176. doi: 10.3389/fnut.2026.1888176 (PMC13639945; doi:10.3389/fnut.2026.1888176)
Supplement: Supplementary file 1 [file Table_1.docx]

Supplementary Material

# 1 Supplementary Methods

## 1.1 Propensity score matching

We first applied nearest neighbor matching with a caliper of 0.1, as it closely mimicked the 1:1 randomization process observed in randomized controlled trials and ensured an optimal balance in baseline covariates between groups. Propensity scores were estimated using multivariable logistic regression. The covariates included age, gender, BMI, education, self-reported physician-diagnosed arthritic conditions or osteoporosis, daily GCC supplement dosage, intake duration, intake frequency, use of other joint- or bone-beneficial supplements, current smoking, regular dairy intake, physical activity, and the use of antihypertensive, antidiabetic, and antihyperlipidemic medications. Next, stabilized IPTW targeting the average treatment effect was applied to adjust for potential confounders between subgroups. To reduce the influence of extreme weights, the weights were winsorized using a 1% trimming threshold. Weighted linear mixed-effects models were subsequently fitted using the IPTW weights to compare symptom-score changes between subgroups. To further assess the robustness of the findings using an alternative weighting approach, OW was also performed using propensity scores estimated from the corresponding subgroup-specific logistic regression models. Under OW, participants in each subgroup were weighted by their estimated probability of belonging to the opposite subgroup, thereby assigning greater weight to participants with greater covariate overlap between the two comparison groups. Weighted linear mixed-effects models using the OW weights were then fitted for the corresponding subgroup comparisons. Covariate balance after matching or weighting was assessed using standardized mean differences.

## 1.2 Assessment of adherence

Two composite adherence scores were developed to assess adherence to the GCC supplement, incorporating intake duration, daily dosage, and frequency of intake. The first was a categorical additive score (cat-add), in which each adherence component was dichotomized and assigned a binary value: intake dosage (half vs recommended dose = 0/1), intake frequency (less than daily vs daily = 0/1), and intake duration (≤3 months vs >3 months = 0/1). These three indicators were summed to yield a total score ranging from 0 to 3, with higher values indicating greater adherence. Participants with a total score of 3 were defined as having high adherence, whereas those with scores of 0-2 were defined as having low adherence. The second was a standardized additive score (z-add) based on z-standardization of dosage, frequency, and duration to ensure comparability across scales. The standardized values were summed to generate a continuous adherence score, with higher values indicating greater adherence. For subgroup analyses, participants with a total score above the median were defined as having high adherence, whereas those with a score at or below the median were defined as having low adherence. For intake frequency, the midpoint of each category was used; for example, 5-6 times per week was recorded as 5.5. Intake dosage was categorized as follows: 1-2 tablets were assigned a value of 0.5, representing half of the recommended dose; 3-4 tablets were assigned a value of 1; and ≥5 tablets were assigned a value of 2, representing a higher-than recommended dosage.

# 2 Supplementary Figures and Tables

## 2.1 Supplementary Figures

**Supplementary Figure 1.** Flowchart of the study. A total of 9,090 individuals who had taken the GCC supplement for at least 1 month were included in the analytic cohort. Among them, 3,896 participants were included in the joint symptom analysis and 2,388 participants in the back pain analysis.

**Supplementary Figure 2.** Directed acyclic graph displaying assumptions about the relationship between variables of interest. Based on the directed acyclic graph, the minimally sufficient adjustment sets (MSAS) are demographic characteristics (age, gender, BMI and educational attainment); medical history of arthritic conditions or osteoporosis; GCC supplement dosage, duration, and frequency; intake of other joint or bone health supplements; lifestyle factors (current smoking, regular dairy intake, and physical activity); and medication use (antihypertensive, antidiabetic, and antihyperlipidemic drugs).

**Supplementary Figure 3.** Usage and intake patterns of the GCC supplement. (A) Self-reported reasons for the GCC supplement intake. (B) Dosage of the GCC supplement intake. (C) Frequency of the GCC supplement intake. (D) Duration of the GCC supplement intake (in log-transformed months).

**Supplementary Figure 4.** Changes in joint discomfort and back pain scores in participants reporting both joint discomfort and back pain. The estimates were obtained from linear mixed-effects models, adjusted for time (pre- and post-use), age, gender, BMI, educational attainment, self-reported arthritic conditions or osteoporosis, daily GCC supplement dosage, intake duration, intake frequency, use of other joint- or bone-health supplements, current smoking, regular dairy intake, physical activity, and use of antihypertensive, antidiabetic, and antihyperlipidemic medications.

**Supplementary Figure 5.** Variable balance before and after propensity score matching and weighting, stratified by dose and duration. Points represent the absolute standardized mean differences of each covariate between different groups. The dotted line shows the prespecified threshold for acceptable covariate balance. Values closer to zero indicate better covariate balance between the two groups.

**Supplementary Figure 6.** Sensitivity analyses of changes in joint discomfort and back pain scores by GCC dosage and duration. Sensitivity analyses evaluating differences in symptom-score changes by dosage and duration were performed using three different statistical approaches: 1:1 caliper matching, inverse probability of treatment weighting (IPTW), and overlap weighting (OW). The estimates were derived from linear mixed-effects models, adjusting for group, time (pre- and post-use), group-by-time interaction and corresponding pre-use symptom scores. To further reduce residual confounding after matching, a double adjustment was applied where necessary.

**Supplementary Table 1.** Questionnaire items for outcome assessment.

| **No.** | **Symptom** | **Questionnaire Item** | **Response Scale** | **Scoring Direction** |
| --- | --- | --- | --- | --- |
| 1 | Joint pain  (before GCC) | Please recall the severity of your joint pain before taking GCC. (0 = no pain; higher scores indicate greater pain severity; 10 = severe pain not relieved even with analgesics) | 0-10 | Higher = worse |
| 2 | Joint stiffness  (before GCC) | Please recall the severity of your joint stiffness before taking GCC. (0 = no stiffness; higher scores indicate greater stiffness; 10 = extremely stiff) | 0-10 | Higher = worse |
| 3 | Joint swelling  (before GCC) | Please recall the severity of your joint swelling before taking GCC. (0 = no swelling; higher scores indicate greater swelling; 10 = severe swelling) | 0-10 | Higher = worse |
| 4 | Joint inflexibility  (before GCC) | Please recall the inflexibility of your joints before taking GCC. (0 = very flexible; higher scores indicate greater inflexibility; 10 = not flexible) | 0-10 | Higher = worse |
| 5 | Joint pain  (after GCC) | Please rate the severity of your joint pain after taking GCC. (0 = no pain; higher scores indicate greater pain severity; 10 = severe pain not relieved even with analgesics) | 0-10 | Higher = worse |
| 6 | Joint stiffness  (after GCC) | Please rate the severity of your joint stiffness after taking GCC. (0 = no stiffness; higher scores indicate greater stiffness; 10 = extremely stiff) | 0-10 | Higher = worse |
| 7 | Joint swelling  (after GCC) | Please rate the severity of your joint swelling after taking GCC. (0 = no swelling; higher scores indicate greater swelling; 10 = severe swelling) | 0-10 | Higher = worse |
| 8 | Joint inflexibility  (after GCC) | Please rate the inflexibility of your joints after taking GCC. (0 = very flexible; higher scores indicate greater inflexibility; 10 = not flexible) | 0–10 | Higher = worse |
| 9 | Back pain  (before GCC) | Please recall the severity of your back pain before taking GCC. (0 = no pain; higher scores indicate greater pain severity; 10 = severe pain not relieved even with analgesics) | 0–10 | Higher = worse |
| 10 | Back pain  (after GCC) | Please rate the severity of your back pain after taking GCC. (0 = no pain; higher scores indicate greater pain severity; 10 = severe pain not relieved even with analgesics) | 0–10 | Higher = worse |

**Supplementary Table 2.** Mean, standard deviation, and distribution-based MCID estimates based on pre-use symptom scores.

| **Symptom** | **Pre-use mean ± SD** | **MCID (0.5 × SD)** |
| --- | --- | --- |
| Joint pain | 3.04 ± 2.44 | 1.22 |
| Joint stiffness | 2.58 ± 2.45 | 1.23 |
| Joint swelling | 1.89 ± 2.37 | 1.19 |
| Joint inflexibility | 3.60 ± 2.98 | 1.49 |
| Back pain | 2.61 ± 2.50 | 1.25 |

Abbreviations: SD, standard deviation; MCID, minimal clinically important difference.

**Supplementary Table 3.** Clinical status of participants with joint symptoms or back pain

| Symptom group | Clinical status | n (%) |
| --- | --- | --- |
| Participants with joint symptoms |  |  |
|  | Osteoarthritis | 951 (24.5) |
|  | Rheumatoid arthritis | 74 (1.9) |
|  | Gouty arthritis | 69 (1.8) |
|  | None of the above joint conditions reported | 2851 (73.5) |
| Participants with back pain | Osteoporosis | 824 (34.5) |

The groups correspond to the participants included in the joint-symptom and back-pain analyses, respectively; clinical diagnoses were based on participants’ reports of previous physician diagnoses. Diagnostic categories were not mutually exclusive.

**Supplementary Table 4.** SRM for pre-use to post-use symptom score changes.

| **Outcome** | **Cases** | **Mean change** | **SD of change** | **SRM** |
| --- | --- | --- | --- | --- |
| Joint pain | 3896 | -1.74 | 2.17 | -0.80 |
| Joint stiffness | 3896 | -1.42 | 2.14 | -0.66 |
| Joint swelling | 3896 | -1.04 | 2.04 | -0.51 |
| Joint inflexibility | 3896 | -0.67 | 3.29 | -0.20 |
| Back pain | 2388 | -1.36 | 2.06 | -0.66 |

Abbreviations: SD, standard deviation; SRM, standardized response means.

**Supplementary Table 5.** Sample sizes before and after propensity score matching and weighting, stratified by dose and duration.

| **Outcome** | **Unweighted sample** | | **Caliper matching** | | **IPTW** | | **OW** | |
| --- | --- | --- | --- | --- | --- | --- | --- | --- |
|  | **Comp** | **Ref** | **Comp** | **Ref** | **Comp** | **Ref** | **Comp** | **Ref** |
| **For joint symptoms** |  |  |  |  |  |  |  |  |
| Recommended and higher dose *vs* half dose | 2609 | 1287 | 1263 | 1263 | 2515 | 1081 | 2301 | 1242 |
| Over 3 to 6 months *vs* 1 to 3 months | 756 | 2557 | 752 | 752 | 705 | 2537 | 749 | 2373 |
| Over 6 months *vs* 1 to 3 months | 583 | 2557 | 580 | 580 | 496 | 2529 | 576 | 2180 |
| **For back pain** |  |  |  |  |  |  |  |  |
| Recommended and higher dose *vs* half dose | 1553 | 835 | 766 | 766 | 1463 | 655 | 1319 | 786 |
| Over 3 to 6 months *vs* 1 to 3 months | 511 | 1519 | 500 | 500 | 455 | 1501 | 503 | 1384 |
| Over 6 months *vs* 1 to 3 months | 358 | 1519 | 346 | 346 | 273 | 1492 | 351 | 1231 |

Comp stands for comparison group, and Ref stands for reference group.

**Supplementary Table 6.** Proportions with symptom-score reduction (≥1-point sensitivity threshold) by dose and duration.

| **Outcome** | **Relief rates based on MCID ≥ 1 (95% CI, %)** | | | | |
| --- | --- | --- | --- | --- | --- |
|  | **All** | **1 month** | **>1 month to ≤3 months** | **>3 months to ≤6 months** | **>6 months** |
| **Half dosage** |  |  |  |  |  |
| Joint pain | 58.82 (56.07 to 61.52) | 49.47 (43.54 to 55.42) | 56.09 (51.86 to 60.24) | 65.93 (59.3 to 72) | 70.64 (64.04 to 76.5) |
| Joint stiffness | 49.34 (46.58 to 52.11) | 40.7 (34.99 to 46.67) | 44.27 (40.11 to 48.5) | 61.06 (54.35 to 67.39) | 61.47 (54.63 to 67.89) |
| Joint swelling | 38.93 (36.26 to 41.66) | 27.02 (22.03 to 32.64) | 34.59 (30.67 to 38.72) | 48.67 (42.02 to 55.37) | 55.5 (48.64 to 62.17) |
| Joint inflexibility | 36.21 (33.59 to 38.91) | 29.47 (24.32 to 35.19) | 36.38 (32.41 to 40.54) | 38.5 (32.18 to 45.21) | 42.2 (35.62 to 49.07) |
| Overall joint symptom | 76.3 (73.86 to 78.58) | 68.77 (62.99 to 74.04) | 73.84 (69.94 to 77.4) | 83.19 (77.52 to 87.69) | 85.32 (79.76 to 89.6) |
| Back pain | 42.63 (39.26 to 46.08) | 33.33 (26.55 to 40.86) | 44.39 (39.36 to 49.52) | 53.33 (45.04 to 61.46) | 37.6 (29.23 to 46.75) |
| **Recommended and higher dosage** | |  |  |  |  |
| Joint pain | 75.66 (73.96 to 77.29) | 67.11 (62.1 to 71.76) | 75.94 (73.53 to 78.19) | 81.7 (78.08 to 84.85) | 74.79 (69.95 to 79.1) |
| Joint stiffness | 66.19 (64.34 to 68) | 57.89 (52.74 to 62.88) | 64.92 (62.28 to 67.47) | 74.72 (70.75 to 78.32) | 67.12 (62.01 to 71.87) |
| Joint swelling | 47.37 (45.44 to 49.31) | 35.26 (30.5 to 40.33) | 46.1 (43.4 to 48.82) | 58.68 (54.34 to 62.89) | 48.22 (43 to 53.47) |
| Joint inflexibility | 40.48 (38.59 to 42.39) | 34.47 (29.75 to 39.52) | 40.7 (38.06 to 43.4) | 42.26 (38.04 to 46.61) | 43.29 (38.17 to 48.55) |
| Overall joint symptom | 89 (87.72 to 90.16) | 83.68 (79.49 to 87.18) | 89.51 (87.71 to 91.07) | 92.83 (90.21 to 94.81) | 87.12 (83.15 to 90.3) |
| Back pain | 68.06 (65.67 to 70.37) | 59.09 (51.42 to 66.36) | 70.75 (67.41 to 73.89) | 73.41 (68.48 to 77.83) | 57.51 (50.88 to 63.89) |

Abbreviation: MCID, minimal clinically important difference.

**Supplementary Table 7.** Summary of self-reported AEs during GCC use.

| **AE** | **Participants (n)** | **Participants (%)** | **Events per 1000 person-month** | **WHO-UMC causality category** |
| --- | --- | --- | --- | --- |
| Constipation | 295 | 3.31% | 7.74 | Possible |
| Diarrhea | 82 | 0.92% | 2.23 | Possible |
| Nausea | 41 | 0.46% | 0.94 | Possible |
| Allergy | 28 | 0.31% | 0.73 | Possible |
| Insomnia | 32 | 0.36% | 0.85 | Unlikely/Unclassified |
| Other/Unspecified | 105 | 1.18% | 2.67 | Unclassified |
| All | 499 | 5.61% | 12.90 | - |

Missing data were noted for 188 participants for adverse events reporting and 1047 participants for intake duration. Percentages and rates were calculated using complete cases only. Abbreviations: AE, adverse event; WHO-UMC, World Health Organization-Uppsala Monitoring Centre.
